# MRI-guided percutaneous thermoablation in combination with hepatic resection as parenchyma-sparing approach in patients with primary and secondary hepatic malignancies: single center long-term experience

**DOI:** 10.1186/s40644-020-00316-z

**Published:** 2020-05-27

**Authors:** Moritz T. Winkelmann, Rami Archid, Georg Gohla, Gerald Hefferman, Jens Kübler, Jakob Weiss, Stephan Clasen, Konstantin Nikolaou, Silvio Nadalin, Rüdiger Hoffmann

**Affiliations:** 1 Department for Diagnostic and Interventional Radiology, University Hospital Tuebingen, Hoppe-Seyler-Straße 3, 72076 Tübingen, Germany; 2 Department of General, Visceral and Transplant Surgery, University Hospital Tuebingen, Tübingen, Germany; 3 Harvard Medical School, Harvard University, Boston, MA USA

**Keywords:** MR-guided intervention, Radiofrequency ablation, Microwave ablation, Hepatic metastases, Hepatic resection, Combination therapy, Percutaneous thermoablation

## Abstract

**Background:**

Combination therapy using hepatic resection (HR) and intra-operative thermal ablation is a treatment approach for patients with technically unresectable liver malignancies. The aim of this study was to investigate safety, survival and local recurrence rates for patients with technically unresectable liver tumors undergoing HR and separate percutaneous MR-guided thermoablation procedure as an alternative approach.

**Methods:**

Data from all patients with primary or secondary hepatic malignancies treated at a single institution between 2004 and 2018 with combined HR and MR-guided percutaneous thermoablation was collected and retrospectively analyzed. Complications, procedure related information and patient characteristics were collected from institutional records. Overall survival and disease-free survival were estimated using the Kaplan-Meier method.

**Results:**

A total of 31 patients (age: 62.8 ± 9.1 years; 10 female) with hepatocellular carcinoma (HCC; *n* = 7) or hepatic metastases (*n* = 24) were treated for 98 hepatic tumors. Fifty-six tumors (mean diameter 28.7 ± 23.0 mm) were resected. Forty-two tumors (15.1 ± 7.6 mm) were treated with MR-guided percutaneous ablation with a technical success rate of 100%. Local recurrence at the ablation site occurred in 7 cases (22.6%); none of these was an isolated local recurrence. Six of 17 patients (35.3%) treated for colorectal liver metastases developed local recurrence. Five patients developed recurrence at the resection site (16.1%). Non-local hepatic recurrence was observed in 18 cases (58.1%) and extrahepatic recurrence in 11 cases (35.5%) during follow-up (43.1 ± 26.4 months). Ten patients (32.3%) developed complications after HR requiring pharmacological or interventional treatment. No complication requiring therapy was observed after ablation. Median survival time was 44.0 ± 7.5 months with 1-,3-, 5-year overall survival rates of 93.5, 68.7 and 31.9%, respectively. The 1-, 3- and 5-year disease-free survival rates were 38.7, 19.4 and 9.7%, respectively.

**Conclusion:**

The combination of HR and MR-guided thermoablation is a safe and effective approach in the treatment of technically unresectable hepatic tumors and can achieve long-term survival.

## Background

Hepatic resection is presently considered the gold standard in potentially curative treatment for patients with hepatic malignancies [1]. However, due to a variety of anatomical and pathological factors, many patients are not candidates for surgical resection, including 70–80% of patients with colorectal liver metastases (CRLM) [2, 3]. High hepatic tumor burden often precludes a curative resection due to insufficient remnant liver function [4]. In order to expand the benefits of hepatic resection to a greater number of patients, a range of strategies have been developed such as neoadjuvant chemotherapy, two-stage hepatectomy, preoperative portal vein embolization and combination of hepatic resection and thermoablation [5–8]. Several groups have reported that combined treatment using hepatectomy and thermoablation may achieve long-term survival rates which are comparable to hepatectomy alone [9–11]. The most common representatives of thermoablation techniques are radiofrequency ablation (RFA) and microwave ablation (MWA), with the latter gaining importance due to its technical advantages as higher intra-tumoral temperatures result in larger ablation zones in a shorter time [12]. MWA is also less susceptible to the heat sink effect, and has recently been reported to be associated with lower local-recurrence rates [13].

For thermoablation in a single procedure, the percutaneous approach is well established with most procedures being performed under CT- or ultrasound guidance [14]. MRI offers an alternative guidance modality, but is presently limited to a small number of specialized institutions. Despite this reduced prevalence, MR-guided procedures offers several clinically valuable advantages over other modalities, including near-real-time MR-fluoroscopy for accurate applicator placement, free selection of imaging planes, MR thermometry for precise delivery of thermal energy, enhanced sensitivity for small target lesions and peri-procedural assessment of the ablation process without requiring application of contrast agents [15–17].

The aim of this study was to investigate safety, survival and local recurrence rates for patients with technically unresectable liver tumors undergoing hepatectomy and separate percutaneous MR-guided thermoablation procedure as an alternative approach to hepatectomy with intraoperative ablation.

## Methods

### Patient population

All patients with liver tumors who received percutaneous thermoablation (RFA or MWA) between June 2004 and July 2018 at a single institution were retrospectively identified from an institutional database. Inclusion criteria included: 1) primary or secondary hepatic malignancy not amenable to hepatic resection alone as determined by an interdisciplinary institutional tumor board, 2) combination therapy using hepatic resection and percutaneous MR-guided thermoablation in a separate procedure, and 3) available follow up imaging. Patients were excluded if percutaneous thermoablation was conducted as therapy for recurrence after prior hepatic surgery, as only planned combination therapy was investigated in this study.

### Percutaneous ablation and surgical resection

Treatment decisions were determined by an interdisciplinary tumor board consisting of representatives from the departments of surgery, internal medicine, radiology, pathology, nuclear medicine and radiotherapy. Decision criteria to determine appropriate therapy included tumor size, number of lesions, anticipated loss of functioning liver parenchyma, presence and degree of cirrhosis and overall state of health. Extent of hepatic resection, lesion assignment to thermoablation and chronological order of resection and ablation were defined in the interdisciplinary tumor board. Hepatic resection was classified according to Brisbane Classification as hemihepatectomy (right or left), sectionectomy (right anterior, right posterior, left medial, left lateral), trisectionectomy (right, left), segmentectomy, bisegmentectomy and atypical resection [18].

One ablation procedure was conducted in a C-shaped, open low-field 0.2 T MRI system (Magnetom Concerto, Siemens Healthineers, Erlangen, Germany). The further 30 procedures were consecutively conducted in a closed bore, whole-body 1.5 T MRI system (Magnetom Espree, Siemens Healthineers). All procedures were conducted in supine position with elevation of the right side if tumors in the right posterior segments were treated [19]. Local anesthesia was injected percutaneously at the puncture site and additional analgesia (piritramide or pethidine) and sedation (midazolam) were administered intravenously during the intervention. In cases of unfavorable tumor position (e.g. subcapsular tumor location), procedures were conducted using general anesthesia. A standardized planning MRI protocol with native sequences was used to confirm tumor number, size, and localization. If tumor visualization was impaired or further hepatic tumor manifestations were suspected, additional diffusion-weighted imaging sequences (DWI) and dynamic contrast-enhanced sequences were acquired after administration of extracellular contrast agent (Gadovist, Bayer HealthCare, Leverkusen, Germany) or hepatocyte-specific contrast agent (Primovist, Bayer HealthCare). Tumor targeting was conducted using steady-state free precession MR-fluoroscopic sequences (BEAT_IRTT) which continuously depicted the applicator path in three dimensions [20]. The interventionalist observed the applicator placement on an in-room LCD-Monitor installed next to the magnet. During thermoablation, the applicator was connected with an extended cable to the generator positioned outside the scanner room. T1- or T2-weighted sequences were used to assess the ablation result as reported in prior work [21]. In the event of an inadequate ablation zone or residual tumor tissue, additional ablation was conducted after repositioning the applicator under MR-fluoroscopy. When the safety margin around the tumor was considered sufficient, the applicator was retracted after tract coagulation. Post-interventional control examination using dynamic contrast-enhanced T1 sequences and a T2 sequence was used to assess the technical success and exclude complications such as hematomas, biliomas, intraperitoneal free fluid or active bleeding. Patients were routinely hospitalized for one night post-ablation and discharged if complete blood count and control ultrasound of the liver were found to be within acceptable limits [20].

### Data collection

The local ethics committee approved the retrospective analysis of patient data. Data concerning demography, tumor entity and use of neo-adjuvant therapies were collected from the interdisciplinary tumor-board records. Tumor size and location were defined by measurements using pre-interventional multiphasic MR-images collected no more than 4 weeks prior to intervention. Complications, duration of hospitalization, administration and timing of systemic therapy, follow-up results and recurrence therapies were collected from institutional medical records and operative/interventional reports. Complications were classified according to the Clavien-Dindo classification [22].

Internal guidelines for follow-up after thermoablation include contrast-enhanced dynamic MRI of the liver 1 month post-procedure, every 3 months thereafter for 1 year and extended to every 6 months thereafter. For data analysis, length of follow-up was defined beginning at the date of the initial procedure and ending at the date of death for deceased patients or at the date of last follow-up visit for surviving patients.

### Statistical analysis

Percentages and mean values were calculated and reported with associated standard deviations. Overall survival and disease-free survival were estimated using the Kaplan-Meier method. Survival data were calculated independently of the censored cases. Group-comparisons were conducted with Student’s t-test. All statistical analysis was performed using SPSS (version 26.0; SPSS Inc., Chicago, IL). *P*-values < 0.05 were considered statistically significant.

## Results

### Patient and tumor characteristics

**Fig. 1:**
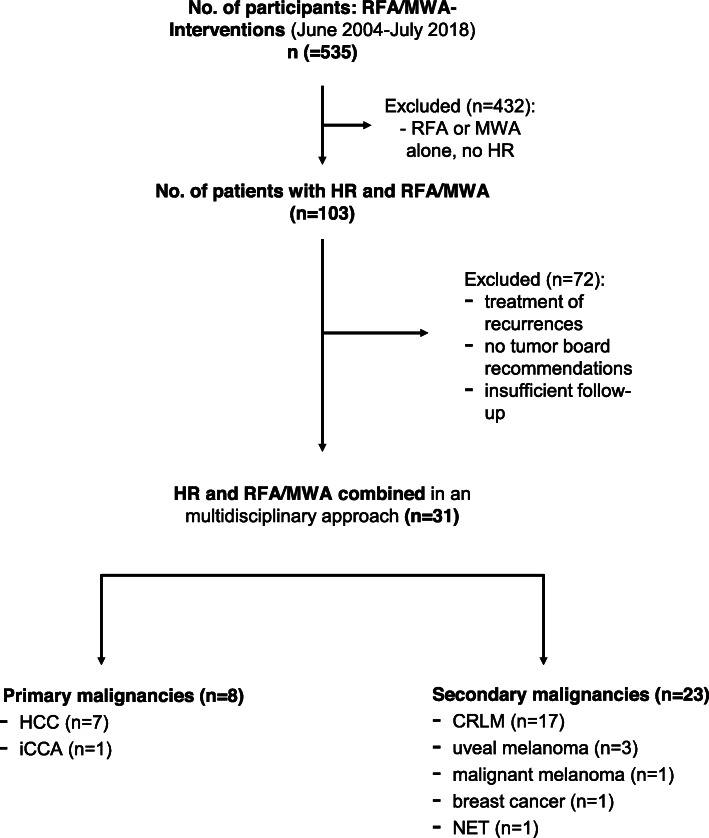
Study flowchart. CRLM = colorectal liver metastases, HCC = hepatocellular carcinoma, iCCA = intrahepatic cholangiocarcinoma, NET = neuroendocrine tumor. Data is given in numbers (n)

A total of 535 patients with primary or metastatic liver tumors were treated with RFA or MWA. Of these, 103 patients underwent additional surgery. Patients who underwent either surgical resection or thermoablation due to a recurrence after intervention were excluded. A total of 32 patients underwent ablation and surgery in combination as recommended by the interdisciplinary tumor board. One patient was excluded due to a lack of follow-up data, resulting in 31 patients included for further analysis (Fig. 1). There were 21 men and 10 women with a median age of 62.8 years ±9.1 years. Of these 31 patients, 17 were patients with colorectal cancer liver metastases (CRLM) and seven with metastases from another tumor entity (Table 1). Seven patients were treated for hepatocellular carcinoma (HCC). A total of 42 lesions with a mean tumor size of 15.1 ± 7.6 mm (range: 4–35 mm) were treated with thermoablation. Twenty-three patients underwent ablation of a single tumor, 6 patients of two tumors, one patient of three tumors and one patient of four tumors, respectively. Fifty-six lesions with a mean tumor diameter of 28.7 ± 23.0 mm (range: 4–90 mm) were surgically resected. Hepatic resections were defined according to the Brisbane classification (Table 2) [18]. Fourteen patients had a single tumor resected, while 17 patients had several lesions resected in one setting (2 lesions in 9 patients and 3 lesions in 8 patients).

**Table 1:** Baseline characteristics

Variables	N (%)/mean ± SD
Age (years)	62.8 ± 9.1
Patients	31
Sex (m/f)	21 (67.8%) / 10 (32.2%)
Number of tumors (ablated/resected)	98 (42/56)
Tumor size of ablated lesions	15.1 ± 7.6 mm
Tumor size of resected lesions	28.7 ± 23.0 mm
Tumor entity	HCC (*n* = 7)
	iCCA (*n* = 1)
	Metastases (*n* = 23)
	Colorectal carcinoma (*n* = 17)
	- Left sided (*n* = 13)
	- Right sided (*n* = 4)
	Uveal melanoma (*n* = 3)
	Malignant melanoma (*n* = 1)
	Breast cancer (*n* = 1)
	Neuroendocrine tumor (*n* = 1)
Concomitant liver cirrhosis	5/31 (16.1%); Child A: *n* = 3, Child B: *n* = 2
Liver function (serum levels):	
GOT/GPT (U/I)	45.2 ± 39.0 / 47.5 ± 43.7
Bilirubin (mg/dl)	0.6 ± 0.3
Quick-Test (%)	96.6 ± 13.9

*HCC* Hepatocellular carcinoma, *iCCA* Intrahepatic cholangiocarcinoma, *TACE* Transarterial chemoembolization

**Table 2:** Surgical procedures according to Brisbane classification [18]

Surgical procedures (liver segments)	N (%)
Right hemihepatectomy	5 (16.1%)
Left hemihepatectomy	5 (16.1%)
Left lateral sectionectomy	1 (3.2%)
Right trisectionectomy	1 (3.2%)
Segmentectomy	3 (9.7%)
Atypical resection	9 (29.0%)
Left hemihepatectomy + atypical resection	3 (9.7%)
Left lateral sectionectomy + atypical resection	3 (9.7%)
Left lateral sectionectomy + segmentectomy + atypical resection	1 (3.2%)

Fifteen of 17 patients treated for CRLM received neoadjuvant chemotherapy before combination therapy. Two patients with HCC received transarterial chemoembolization (TACE) before the ablation procedure. In one patient with HCC, combination therapy was conducted for bridging before liver transplantation, which was conducted 6 months after ablation.

### Procedures

**Fig. 2:**
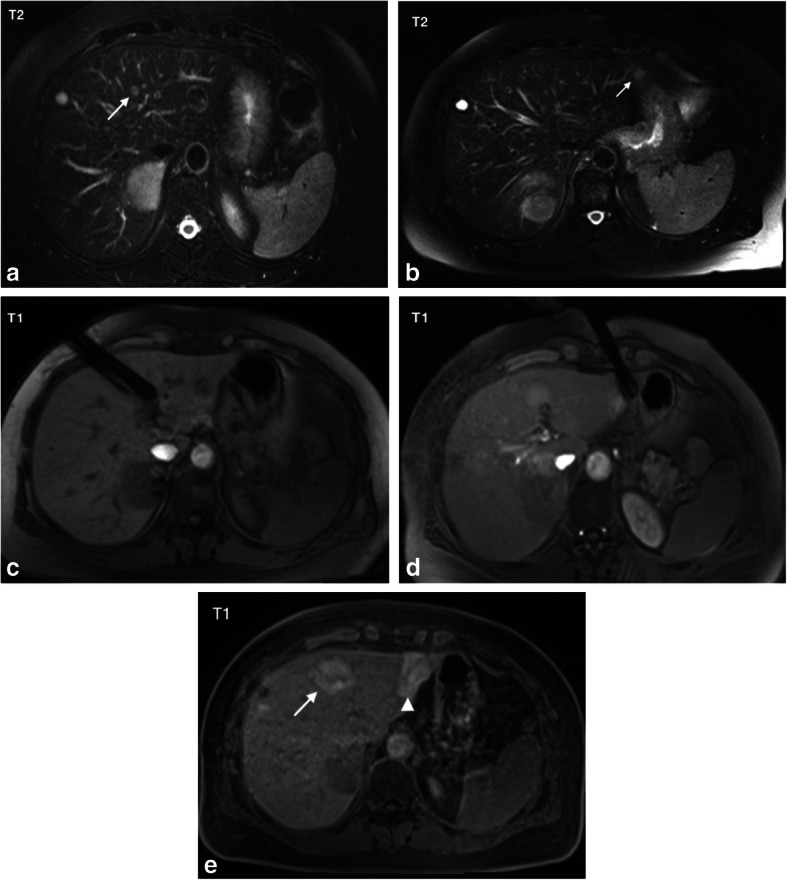
62-year old female patient with hepatic metastases from a uveal melanoma in both liver lobes. According to tumor-board decision, right hemihepatectomy was planned after thermoablation of a metastasis in the left liver lobe. Pre-interventional T2-weighted TSE imaging (**a**) shows the target lesion planned for thermoablation. However, intra-procedural planning imaging (**b**) reveals a new small lesion subcapsular in segment 2 (arrow). Both tumors could be treated during the same procedure as depicted by intra-interventional T1-weighted sequences (**c** and **d**) showing two radiofrequency antennas targeting both target tumors. T1-weighted control imaging shows the T1 hyperintense ablation zones covering both target tumors (**e**). Right hemihepatectomy was conducted 1 month after ablation

Radiofrequency ablation was performed in 24 patients and MWA in 7 patients. All procedures were conducted under MR-guidance and technical success was achieved in all ablation procedures. The average procedure duration including acquisition of planning sequences, applicator positioning (mean number of needle positions: 3.0 ± 1.3), therapy monitoring and post-interventional control imaging was 4.8 h. Procedures with MWA were significantly faster than procedures with RFA (3.3 vs. 5.3 ± 1.4 h) (*p* < 0.001). Fourteen patients received thermoablation before surgical resection (mean interval 0.9 ± 0.6 months) and 17 patients received thermoablation after surgery (mean interval 2.0 ± 3.1 months). In one patient with hepatic metastases from a uveal melanoma, a peritoneal carcinosis was diagnosed during atypical hepatectomy; consequently, planned thermoablation was postponed and systemic therapy administered. After a positive response to chemotherapy, thermoablation was then conducted 13 months after the initial surgery. Excluding this case, the mean period between resection and ablation was 1.3 ± 0.9 months. In four patients, a total of six additional lesions were detected by intra-procedural planning imaging. These new lesions with mean diameter of 7.2 ± 3.2 mm (range: 4–11 mm) were ablated in the same procedure (Fig. 2a-e). Length of hospital stay after thermal ablation was 1.8 ± 1.1 days (range: 1–5 days). Length of hospital stay after surgery was 12.8 ± 6.2 days (range: 7–37 days).

### Complications

Ten patients suffered complications after surgery. Grade 2 complications in which pharmacological treatment was necessary were observed in 6 patients and included ascites, capillary leak, infection, pneumonia and hypertension. One patient developed a hematoma of the abdominal wall which necessitated treatment without general anesthesia (Grade 3a). Following surgery, one patient had wound infection (Grade 3a). A subhepatic abscess was observed in two patients and re-laparotomy had to be performed (Grade 3b). No mortality has occurred.

No complications were observed after thermoablation. One patient developed acute conjunctivitis after RFA resulting in a post-interventional hospitalization period of 5 days; however, this event was determined not to be related to the thermoablation procedure.

### Recurrence

Overall, recurrence was observed in 26 patients. One of seven patients (14.2%) with HCC and 6/24 (25%) with hepatic metastases (all treated for CRLM) developed local recurrence at the ablation site. Overall local recurrence rate at the ablation site was 22.6% on a per-patient-basis and 16.6% on a per-tumor-basis. Two of 6 patients (33%) with local recurrence of a CRLM had a primary right-sided colon carcinoma. None of the patients with local recurrence at the ablation site developed an isolated local recurrence (one patient had additional recurrence at the resection margin, three patients had an extrahepatic recurrence and five patients developed new manifestations in the remaining liver). The mean diameter of the ablated tumors with local recurrence (22.9 ± 7.3 mm) was significantly larger than in tumors without local recurrence (13.2 ± 6.0 mm; *p* = 0.017). None of the patients treated with MWA developed local recurrence during follow-up.

Recurrences at the surgical resection site were observed in 5 patients (16.1% on a per-patient-basis, 8.9% on a per-tumor-basis), including one patient with recurrence at both the resection area and the ablation zone. A total of 18 patients (58.0%) developed non-local hepatic recurrences, and a total of 11 patients (35.5%) were found to have extrahepatic recurrence during follow-up. During follow- up, nine patients underwent percutaneous thermoablation for 16 non-local recurrences and two patients underwent a second resection due to new hepatic tumors.Table 3 summarizes the cases with local recurrence at the ablation zone and the resection site and states the therapy after local recurrence. During follow- up, nine patients underwent percutaneous thermoablation for 16 non-local recurrences and two patients underwent a second resection due to new hepatic tumors.

**Table 3:** Cases with local recurrence at the ablation zone and resection site including therapy after recurrence

Case	Recurrence location	Target tumor	Non-local recurrence during follow-up	Initial Therapy after local recurrence	Further therapies during follow-up
1	RS	HCC	yes	SIRT	Systemic therapy
2	AZ + RS	CRLM	yes	Systemic therapy	–
3	RS	HCC	yes	Systemic therapy	–
4	AZ	CRLM	yes	SIRT	Systemic therapy
5	AZ	CRLM	yes	Systemic therapy	–
6	AZ	CRLM	yes	Hepatic resection	Systemic therapy
7	AZ	CRLM	yes	Thermal ablation	Thermal ablation
8	AZ	CRLM	yes	Thermal ablation	–
9	RS	HCC	no	SIRT	Systemic therapy
10	RS	CRLM	yes	Systemic therapy	–
11	AZ	HCC	yes	Thermal ablation	Thermal ablaton (2x)

*AZ* Ablation zone, *RS* Resection site, *SIRT* Selective internal radiation therapy, *CRLM* Colorectal liver metastases, *HCC* Hepatocellular carcinoma

### Survival

**Fig. 3:**
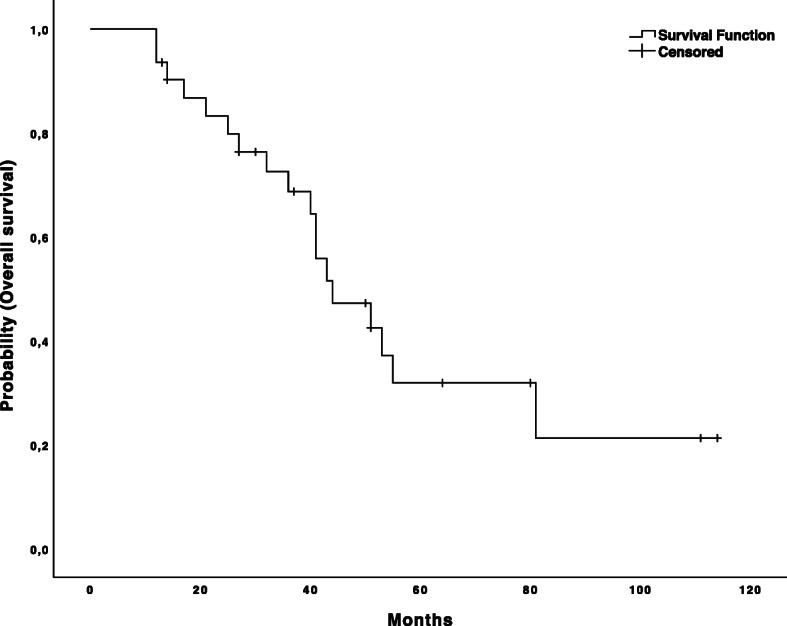
The Kaplan-Meier overall survival curve. The Kaplan-Meier curve for overall survival after combined therapy. The starting point for calculation is the date of first treatment (ablation or resection) within the combined therapy

**Fig. 4:**
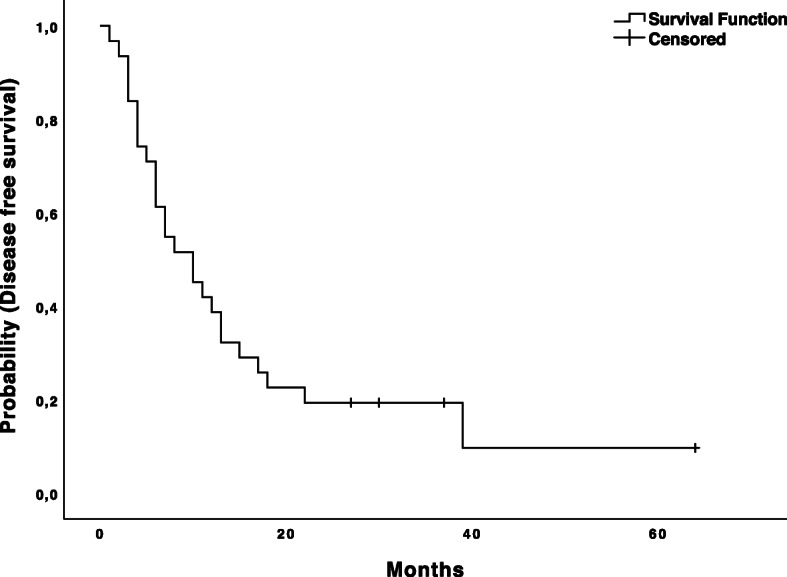
The Kaplan-Meier disease-free survival curve. The Kaplan-Meier curve for disease-free survival after combined therapy. The starting point for calculation is the date of first treatment (ablation or resection) within the combined therapy

The median length of follow-up was 43.1 ± 26.4 months. At the time of last follow-up, 11 patients were alive (five of those without evidence of disease), 18 patients died, and three were lost to follow-up. The median survival time was 44.0 ± 7.5 months and 1, 3, and 5-year overall survival (OS) rates were 93.5, 68.7 and 31.9%, respectively (Fig. 3). The median disease-free survival time was 10.0 ± 2.3 months. The 1, 3, and 5-year disease-free survival rates after thermal ablation and hepatic resection were 38.7, 19.4 and 9.7%, respectively (Fig. 4).

## Discussion

The combination of surgical resection and thermoablation is a common approach to extend resectability in patients with hepatic malignancies. Several studies describe this approach, which employed a combination of resection and immediate intraoperative ablation [23, 24]. The approach described in this work differs in that percutaneous, MR-guided thermoablation was conducted separately from the hepatic resection. Although the duration of hospitalization after percutaneous thermoablation was relatively short, this alternative approach is associated with a second procedure, and thus generally with a second hospitalization for the patient. Nevertheless, the motivation for this approach which has been conducted at our institution for over 10 years is based on the potential advantages of MR-guided thermoablation. MRI-guided procedures offer high soft tissue contrast and enables an immediate assessment of the ablation process without the administration of contrast medium, as the ablation zone can be seen as a T1 hyperintense area, whereas the target tumor is typically hypointense on T1 weighted imaging [21]. In contrast, with CT as a guidance modality, both the ablation zone and the target tumor appear hypodense in unenhanced imaging making a reliable differentiation between tumor and the ablation zone difficult [25]. Furthermore, the ablation process causes the formation of gas at the ablation zone, so that the assessment of the ablation process is challenging using ultrasound guidance [26]. MR fluoroscopy with free angulation of the imaging slices allows real-time monitoring and reconfiguration of applicator positioning, allowing tumors in challenging anatomical locations to be more safely reached [20, 27]. The latter can be of particular relevance for the patients in our study if initial right-side hemihepatectomy causes a shift of the remaining left liver lobe so that the access is limited and applicator positioning is challenging. A further advantage of MRI as guidance modality is the high sensitivity in detection of small liver lesions [28]. Several studies have shown that MRI has a higher sensitivity in detection of small liver lesions in comparison to CT, so that pre-operative acquisition of hepatic MRI can lead to a significant change in the treatment plan [29]. In four of our study patients, planning imaging at the beginning of the intervention revealed six new and therefore suspicious lesions, which could be successfully treated in the same procedure. Due to the small diameter of these new lesions ranging from 4 to 11 mm, it is questionable if they would have been detected during planning imaging using other modalities. Despite these technical advantages of MRI as guidance modality, reduction of procedure durations is of particular interest to establish MR-guided thermoablation in clinical routine. In this context, MWA is a promising ablation technique as significantly shorter procedure durations can be achieved in comparison to RFA [13].

A comparison of the survival and recurrence data with earlier studies regarding resection combined with intra-operative thermoablation is difficult as available study results show heterogeneous cohorts and results. Qiu et al. reported the long-term outcome after hepatic resection and ultrasound-guided intraoperative radiofrequency ablation in 112 patients with HCC or hepatic metastases [30]. The 3 and 5-year survival rates in the HCC group were 32.5 and 12.5% and 50 and 19.4% in the group with liver metastases, respectively. Recurrence-free survival in the same study after 1 and 3 years in the HCC-Group was 52.5 and 22.5% respectively and 58.3 and 23.6% in the liver metastases group. Eisele et al. reported their results on 22 patients with primary and secondary hepatic malignancies who underwent hepatic resection combined with intraoperative radiofrequency ablation with a local recurrence rate at the ablation site of 32% on a per-patient basis. The 1- and 3-years overall survival rate of this study was reported as 77 and 41%, respectively [31]. Moreover, this study summarized the available literature and reported a high variation of overall survival rates ranging from 43 to 92% after 1 year. Although the survival rates in our study were above average, a relatively high local recurrence rate of 21.9% is striking. A previous study by Rempp et al. investigating MR-guided percutaneous RFA of primary and secondary liver tumors as a monotherapy showed a local recurrence rate of 8.6% [20]. As discussed by Sasaki et al., this effect may be explained by a selection bias, as patients undergoing combination therapy have different baseline characteristics compared to patients who undergo only one therapy, which may affect recurrence rates and observed outcome [11]. A potential indicator for unfavorable tumor characteristics in the patient cohort with local recurrence is the simultaneous high rate of non-local recurrence, as all patients with local recurrence developed new intra- or extrahepatic metastases. Furthermore, we observed an exceptionally high rate of local recurrence in the CRLM group (6 of 17 patients). Potential risk factors for local recurrence and poor oncological outcomes may include mutations such as BRAF and KRAS or right-sided colon cancer [32–34].

However, mutation analysis was not available in most cases of this retrospective study, so that an assessment of the effect of KRAS and BRAF mutations on local recurrence rates was not possible. Nevertheless, larger ablation zones with a larger safety margin should be achieved in this patient cohort, especially when larger tumors are treated.

The major limitations of this study are its retrospective design and the limited patient cohort with a high heterogeneity regarding tumor entities. Several factors which may have influenced the outcome, such as mutation status, could not be retrospectively collected. Furthermore, technical aspects such as the ablation technique may affect the ablation result but were not in the focus of this study due to the limited patient number.

## Conclusions

In conclusion, the combination of hepatic resection and MR-guided thermoablation is a safe and effective approach in the treatment of technically unresectable hepatic tumors and can achieve long-term survival. MR-guidance may have an effect on the course of treatment due to the high sensitivity regarding detection of small hepatic tumors. Nevertheless, larger prospective studies are necessary to evaluate the influence of tumor characteristics and ablation techniques on patient outcome.

## Supplementary information

**Additional file 1.**

## Data Availability

A source data table is available.

## Abbreviations

BRAF: V-raf murine sarcoma viral oncogene homolog B1
iCCA: Intrahepatic Cholangiocarcinoma
CRLM: Colorectal liver metastases
CT: Computed tomography
DWI: Diffusion-weighted imaging
HCC: Hepatocecellular carcinoma
HR: Hepatic resection
KRAS: Kirsten rat sarcoma viral oncogene homolog
LCD: Liquid crystal display
MRI: Magnetic resonance imaging
MWA: Microwave ablation
NET: Neuroendocrine tumor
OS: Overall survival
RFA: Radiofrequency ablation
SIRT: Selective internal radiation therapy
TACE: Transarterial chemoembolization
TSE: Turbo spin echo
